# The quality control of measured data

**DOI:** 10.1155/S1463924683000383

**Published:** 1983

**Authors:** L. Leisztner, P. Barna

**Affiliations:** Institute of Forensic Science Pf.: 314/4 Budapest H-1903 Hungary


Journal of Automatic Chemistry, Volume 5, Number 3 (July–September 1983), pages 155–156

### The quality control of measured data

**L. Leisztner and P. Barna**

*Institute of Forensic Science, H-1903 Budapest Pf.: 314/4, Hungary*

In digital, computerized analytical measurement raw data signal samples are collected at a given frequency. In two-dimensional analytical methods the analytical information is produced by different algorithms, which can be the integrated peak area, the height of the peak, or the extreme values of the measured signal (for example the retention time in chromatography). The analytical information thus produced is always biased since an element of 'noise' is inevitable [1 and 2]. Recent advances in microelectronics, coupled with cheaper technology, promise a new generation of analytical instruments. There are several analytical areas where microprocessors offer new, or wider, capabilities [3], for example qualification of measurements by estimating the standard deviation of the random error term during measurement. The application of constant signal-to-noise ratio (SNR) strategy is a way of determining the efficiency of a measurement procedure [4]. However, there is no previously reported method for calculating standard deviation of random errors of the measured signal samples generated by two-dimensional analytical methods. This paper describes such a method.

The signal samples of an analytical measurement contain signal and noise components from different sources. Random errors cannot be determined directly; however, the standard deviation of the random errors can be estimated as follows:

$$Y_i = \bar{Y}_i + \mu_i \qquad (1)$$

where $Y_i$ is the measured signal sample;

$\bar{Y}_i$ is the part of the signal without random errors; and

$\mu_i$ is the random error term.

The difference test is applicable to successive random error terms, so equation (2) below is suitable for estimating standard deviation:

$$D^2 = \frac{\sum_{i=1}^{n} (\mu_i - \mu_{i+1})^2}{2(n-1)}. \qquad (2)$$

The error term $\mu_i$ cannot be determined directly so $(\mu_i - \mu_{i+1})$ is approximated with $Y_i$ and $\overset{\Delta}{Y}_i$, where $\overset{\Delta}{Y}_i$ is the 'slightly smoothed' value of the measured $Y_i$. The characteristic constant $a$ of the smoothing can be defined as:

$$a = \frac{\bar{Y} - \overset{\Delta}{Y}}{\bar{Y} \cdot Y}. \qquad (3)$$

Equation (1) with approximation of $(\mu_i - \mu_{i+1})$ gives:

$$(\mu_i - \mu_{i+1}) = [Y_i - \bar{Y}_i - (Y_{i+1} - \bar{Y}_{i+1})]. \qquad (4)$$

Using equation (3):

$$(\mu_i - \mu_{i+1}) = \frac{1}{1-a}(Y_i - \overset{\Delta}{Y}_i - Y_{i+1} + \overset{\Delta}{Y}_{i+1}). \qquad (5)$$

The smoothed $\overset{\Delta}{Y}_i$ is calculated according to the following equation:

$$\overset{\Delta}{Y}_i = \frac{\sum_{j=-M}^{+M} C_j Y_{i+j}}{\sum_{j=-M}^{+M} C_j}. \qquad (6)$$

The smallest $M$ value possible has been used: $M = 1$, with $C_j = 1$ for every $j$. The condition for using equation (5) can be formulated as:

$$|\bar{Y}_i - \bar{Y}_{i+1}| \ll |\mu_i - \mu_{i+1}|. \qquad (7)$$

So the sampling frequency of the data acquisition must be high enough to satisfy this. Using equations (2) and (5) to estimate standard deviation gives:

$$D = \frac{\sqrt{\sum_{i=1}^{n} (Y_i - \overset{\Delta}{Y}_i - Y_{i+1} + \overset{\Delta}{Y}_{i+1})^2}}{(1-a)\sqrt{2}\sqrt{n-1}}, \qquad (8)$$

where

$$\overset{\Delta}{Y}_i = \frac{Y_{i-1} + Y_i + Y_{i+1}}{3}.$$

Enke and Nieman [5] examined the influence of digital filtering on normally and uniformly distributed noises. On the basis of their graphical data, a value of $a$ for $M = 1$ was estimated: it should be much smaller than 1. To find a more precise value for $a$ 7000 signal samples with normally distributed noise were generated; the value was 0·0059. As $a \ll 1$, equation (8) can be rewritten in a more usable form:

$$D \simeq D' = \frac{\sqrt{\sum_{i=1}^{n} (Y_i - \overset{\Delta}{Y}_i - Y_{i+1} + \overset{\Delta}{Y}_{i+1})^2}}{\sqrt{2}\sqrt{n-1}}. \qquad (9)$$

All the variables in the above equation can be either measured or calculated. To check equation (9) normally distributed noise was superimposed on uniform signal samples. Its standard deviation was $S = 10624$. $D'$ was calculated from equation (9) and the value was found to be 10880. Correcting the value of $D'$ by $1 - a$ gives:

$$D = \frac{10880}{1 - 0{\cdot}0059} = 10945.$$

For the $D$ and $S$ values the $F$ test shows no significant difference—it seems that neglecting the correction with $a$ does not cause a significant error.

To test the applicability of equation (9) to analytical signals it was necessary to simulate chromatographic measurements with a computer-generated model to determine the standard deviation of the error. An HP 21MX computer was used for the simulation. The chromatographic peaks and noise were generated by the method described by Chesler and Cram [6]. Different peak shapes and different SNRs with known standard deviation were generated at a sampling frequency was 8 Hz. Table 1 shows peak width, SNR, the mean of the standard deviation of the noise for 10 parallels, the standard deviation of the noise generation, the mean value and the standard deviation of 10 parallels of $D'$ calculated according to equation (9) for the whole chromatogram. It also shows the values for the signal samples, which belong to the chromatographic peak. The data demonstrate that there is no significant difference between the generated and the estimated values of the standard deviation of the error.

*Table 1. Estimated standard deviation of the random errors of signal samples.*

| $W$ | SNR | $S$ | $S_g$ | $D'_1$ | $S_{D'1}$ | $D'_2$ | $S_{D'2}$ |
|---|---|---|---|---|---|---|---|
| 4 | 10 | 398·9 | 23·5 | 412·7 | 22·3 | 411·6 | 116·8 |
| | 100 | 39·9 | 3·6 | 41·2 | 3·4 | 42·8 | 11·5 |
| | 500 | 7·98 | 0·73 | 8·16 | 0·57 | 8·16 | 1·68 |
| | 2000 | 1·99 | 0·13 | 1·80 | 0·08 | 2·29 | 0·30 |
| | 10 | 199·5 | 11·8 | 208·3 | 7·2 | 204·4 | 43·8 |
| | 100 | 20·0 | 1·79 | 21·0 | 1·90 | 21·3 | 4·50 |
| | 500 | 4·02 | 0·36 | 4·10 | 0·21 | 4·30 | 0·66 |
| | 2000 | 1·00 | 0·06 | 0·85 | 0·05 | 1·07 | 0·18 |
| 16 | 10 | 99·6 | 5·90 | 109·9 | 4·30 | 86·4 | 12·9 |
| | 100 | 9·96 | 0·89 | 10·4 | 0·66 | 10·7 | 1·30 |
| | 500 | 1·99 | 0·18 | 1·93 | 0·11 | 2·12 | 0·28 |
| | 2000 | 0·50 | 0·03 | 0·50 | 0·02 | 0·63 | 0·06 |
| 32 | 10 | 48·7 | 2·90 | 53·2 | 3·20 | 55·5 | 10·6 |
| | 100 | 4·87 | 0·31 | 5·20 | 0·34 | 5·10 | 1·10 |
| | 500 | 0·97 | 0·09 | 1·03 | 0·06 | 1·12 | 0·07 |
| | 2000 | 0·24 | 0·01 | 0·35 | 0·01 | 0·40 | 0·02 |

*Key to table 1.* $W$ = peak width (s); SNR = signal-to-noise ratio; $S$ = mean standard deviation of generated noise for 10 parallels; $S_g$ = standard deviation of the noise generation; $D'_1$ = mean $D'$ of 10 parallels for the whole chromatogram; $S_{D'1}$ = standard deviation of $D'_1$; $D'_2$ = mean $D'$ of 10 parallels for the signal samples which belong to the chromatographic peak; and $S_{D'2}$ = standard deviation of $D'_2$. The values of $D'_1$ and $D'_2$ were calculated according to equation (9).

### References

1. Smit, H. C. and Walg, H. L., *Chromatographia*, **8** (1975), 311.
2. Duursma, R. P. J. and Smit, H. C., *Analytica Chimica Acta*, **133** (1981), 67.
3. Stockwell, P. B. and Telford, I., *Chromatographia*, **13** (1980), 665.
4. Darland, E. J., Lerol, G. E. and Enke, C. G., *Analytical Chemistry*, **52** (1980), 714.
5. Enke, C. G. and Nieman, T. A., *Analytical Chemistry*, **48** (1976), 705A.
6. Chesler, S. N. and Cram, S. P., *Advances in Chromatography* (1971), 1.

### Correspondence:
### 'Micro Program of the Month'

We read your editorial in Volume 5, Number 1 of the *Journal of Automatic Chemistry* with great interest. In the second paragraph you mention your difficulty in dealing with the applications of computers because of the widely varying range of experience of your readers, and you offer to deal with readers' problems.

We would like to make a proposal which might address part of that range, namely that you run a 'Micro Program of the Month' series.

What we had in mind is a feature in each issue for a program (or programs) in use in the author's laboratories, with brief explanatory notes. We felt that if the tone were relatively informal it could provide a useful forum to help your readership exchange views and build up, simply and inexpensively, libraries of small programs from other readers' ideas and routines.

We have discussed the idea with a few colleagues in the clinical chemistry world and they would welcome such a series. We believe we could support it with contributions of programs that we have written for use in our own laboratories until such time as it attracted suitable contributions from the rest of your readership.

We will be glad to discuss the idea with you and your Editorial Board in more detail if you believe it might be useful.

**P. W. N. Gordon**
*Biochemistry Department, St. Andrew's Hospital, Billericay, Essex, UK*

**and G. S. Challand**
*Department of Chemical Pathology, Charing Cross Hospital, London W6 8RF*

### THE PITTSBURGH CONFERENCE

*5–10 March 1984 at Atlantic City, New Jersey, USA*

The call for Papers for the 35th Pittsburgh Conference was issued during June. Abstracts are due by 15 August—those received after this date are not guaranteed consideration for inclusion in the technical programme. The following symposia have been organized:

Spotlight on chromatography
Analytical techniques using supercritical fluids
Advanced light sources
Microprobe techniques as applied to organic materials
New techniques in electroanalytical chemistry
New opportunities in mass spectrometry
Sample introduction for plasma and flames: how can we do it better?
Integrating software into laboratory systems
Polymer characterization
Industrial hygiene monitoring
New horizons in nuclear magnetic resonance
The really sensitive techniques
ASTM E-42—Industrial applications of surface analysis
Pittsburgh analytical chemistry award
Pittsburgh spectroscopy award
Dal Nogare award symposium
The Williams-Wright industrial spectrocopist award

The symposium 'Spotlight on chromatography' will feature three invited symposia and an evening discussion group sponsored by the Pittsburgh Conference and organized by the International Meeting on Capillary Chromatography.

General papers are not restricted to symposia topics: they can be contributed in all areas of the disciplines of analytical chemistry and applied spectroscopy.

*The Continuing Education Programme*

1984's programme will contain, on 9 and 10 March, a short course on 'Hardware and software solutions to laboratory data management problems' (directed by Frank W. Plankey, Jr.), Mini short courses also on 9 and 10 March; and workshops on applications of personal computers on 8 March.

*Contacts*

Those wishing to read a paper should write to Mrs Linda Briggs (Department J-212) and enquiries about reserving space should be directed to George Vassilaros, *Pittsburgh Conference, 437 Donald Road, Pittsburgh, Pennsylvania 15235, USA.*

